# *In vitro* and *in vivo* evaluation of humanized monoclonal antibodies targeting pneumolysin

**DOI:** 10.1128/spectrum.00277-25

**Published:** 2025-07-25

**Authors:** Lijun Song, Huixiu Hu, Jing Huang, Houfeng Li, Ying Zhang, Ning Ma, Zheming Song

**Affiliations:** 1 Department of Anesthesiology, Qingpu Branch of Zhongshan Hospital Affiliated to Fudan University 668070, Shanghai, China; 2 Graduate School, Hebei North University 261761, Zhangjiakou, Hebei, China; 3 Graduate School, Wannan Medical College 74649, Wuhu, Anhui, China; 4 Department of Clinical Laboratory, 905th Hospital of PLA, Shanghai, China; 5 Department of Anesthesiology, 905th Hospital of PLA, Shanghai, China; Meijo University, Nagoya, Japan

**Keywords:** *Streptococcus pneumoniae*, pneumolysin, monoclonal antibody, humanization

## Abstract

*Streptococcus pneumoniae* is a gram-positive bacterium that causes a variety of diseases, including otitis media, sinusitis, pneumonia, bacteremia, and meningitis. While pneumococcal conjugate vaccines have reduced disease burden globally, serotype replacement affects the effectiveness of the vaccine, and *S. pneumoniae* has become resistant to a variety of antibiotics. Infections caused by antibiotic-resistant strains can interfere with the treatment of associated diseases. Monoclonal antibody therapy has emerged as a promising solution to the clinical problems caused by refractory *S. pneumoniae*. Enzyme-linked immunosorbent assay was used to detect antigen-antibody affinity. The anti-hemolytic activity of the antibody was comprehensively evaluated by *in vitro* anti-hemolytic assay and *in vivo* mouse assay. The blood concentration-time change of the antibody was evaluated by pharmacokinetic assay. Humanized monoclonal antibody (2E5zumab) was found to have high affinity for pneumolysin (PLY). *In vitro* anti-hemolysis experiments demonstrated that the antibody could effectively neutralize PLY. *In vivo* experiments indicated that the survival rate of *S. pneumoniae*-infected mice was significantly improved by the antibody (*P* < 0.01). Pharmacokinetic studies revealed that the antibody possessed a long half-life and high bioavailability, which were essential for sustained resistance to the toxin. In summary, the humanized antibody (2E5zumab) was successfully prepared, and the monoclonal antibody showed significant pharmacodynamic effects within *in vivo* mouse models and *in vitro* experiments.

**IMPORTANCE:**

*Streptococcus pneumoniae* is a leading bacterial cause of a wide range of infections, including otitis media, community-acquired pneumonia, sepsis, and meningitis. Pneumococcal disease has become a major public health problem worldwide. In this study, we developed a humanized monoclonal antibody (2E5zumab). *In vitro* functional assays demonstrated that the antibody was effective in neutralizing PLY, and *in vivo* studies showed that the antibody had a strong effect against pneumococcal infections. Therefore, the present study provides a new humanized anti-PLY antibody that is important for the development of therapeutic interventions against pneumococcal infections.

## INTRODUCTION

Pneumococcal disease has become a major public health problem worldwide (1, 2). *Streptococcus pneumoniae* is a gram-positive bacterial pathogen that colonizes the mucosal surfaces of the nasopharynx and upper respiratory tract of the host, causing otitis media, pneumonia, sepsis, and meningitis, especially in children and the elderly (3–5).

Vaccination plays a crucial role in preventing pneumococcal disease (6, 7). While pneumococcal conjugate vaccines target specific serotypes, their widespread use may drive serotype replacement, leading to an increased incidence of diseases caused by non-vaccine serotypes (8, 9). The protein vaccine is not limited by serotype but is still in the research phase (10, 11). Antibiotics are the primary treatment for pneumococcal infections; however, due to the overuse of antibiotics, *S. pneumoniae* has developed not only resistance to single antibiotics but also multi-drug resistance (12, 13). While antibiotics can inhibit or have a bacteriostatic effect on *S. pneumoniae*, they are ineffective against the toxins released during bacterial lysis. Antibody therapies are increasingly being utilized to address a broader spectrum of human diseases, including metabolic diseases, hematologic disorders, and infectious diseases (14–16). Monoclonal antibodies (mAbs) are characterized by their high specificity and potency, primarily serving diagnostic and therapeutic purposes (17). It is widely used in tumors and autoimmune diseases, and mAbs have become the main treatment for some diseases (18). However, antibody engineering may increase the risk of immunogenicity in patients, including the generation of anti-drug antibodies (ADAs). ADAs can lead to diminished efficacy of therapeutic antibodies, accelerated clearance through the formation of immune complexes with therapeutic antibodies, compromised drug safety and effectiveness, and even the discontinuation of clinical trials (19). However, the immunogenicity of antibodies currently under investigation can be mitigated to some extent once they are humanized (14).

Pneumolysin (PLY), the major protein virulence factor of *S. pneumoniae* produced by virtually all strains of clinical isolates, binds to cell membrane cholesterol and lyses cells by oligomerizing multiple toxin monomers to form large pores in the cell membrane (20). PLY exhibits cytotoxic activity for virtually every cell type in the body, including epithelial and immune cells, etc. (21–23). Because PLY is conserved between *S. pneumoniae* strains and serotypes, it has been extensively studied as a therapeutic target (24). Currently, research on neutralizing antibodies against PLY is still in its infancy, and there are no antibodies specifically directed against PLY that are widely available or approved for use in the clinic.

In this study, we developed a humanized mAb (2E5zumab) with high affinity for PLY and verified the efficacy of the antibody both *in vivo* and *in vitro*. These studies provide new insights into anti-pneumococcal mAb protection.

## RESULTS

### *S. pneumoniae* causes invasive infection, and its toxins cause red blood cell (RBC) lysis and mouse death

**Fig 1:**
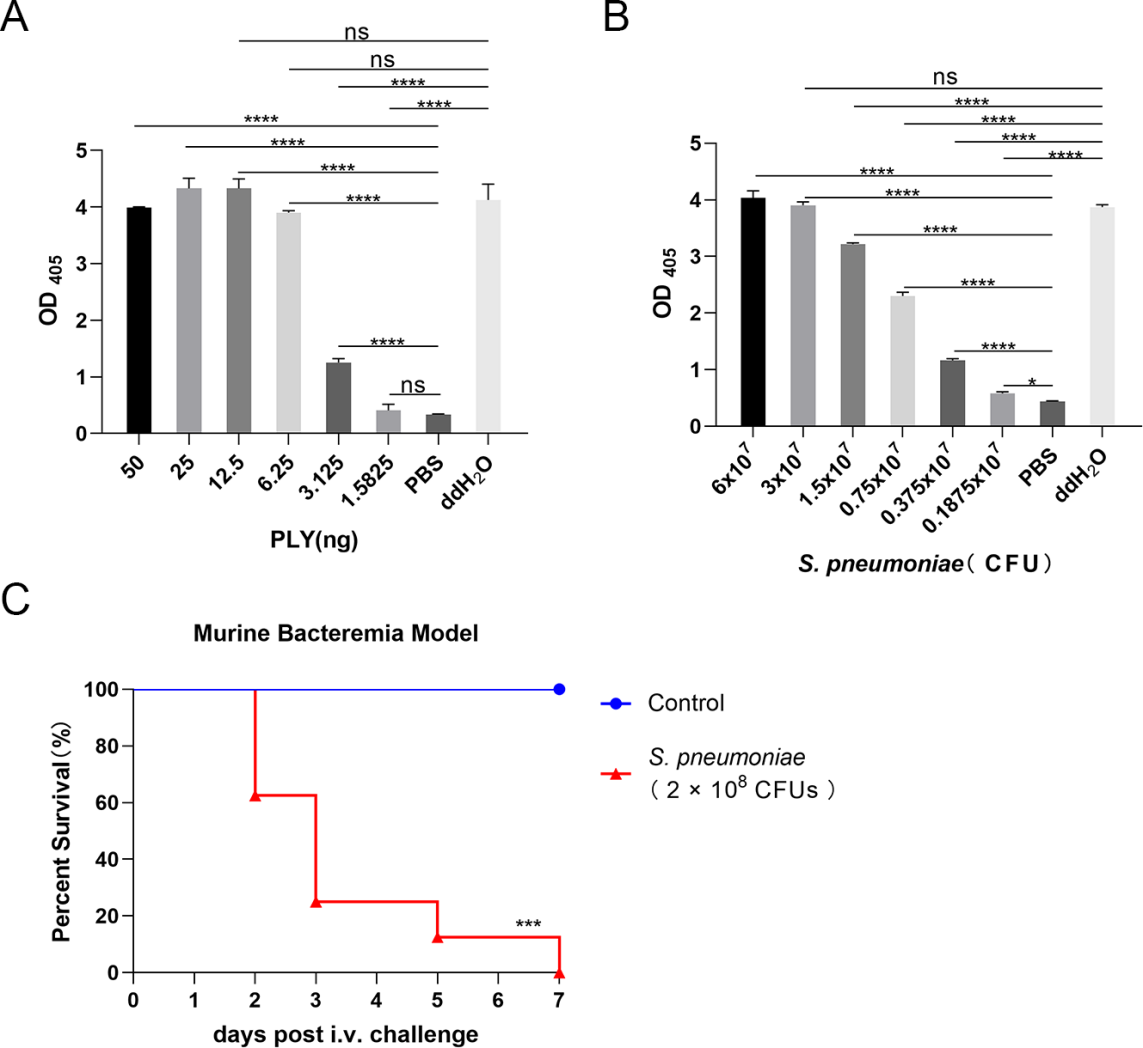
Hemolytic activity of PLY and *S. pneumoniae*. (**A**) Hemolysis of RBCs induced by PLY. (**B**) Hemolysis of RBCs by *S. pneumoniae*. The minimum complete hemolytic dose was 3 × 10^7^ CFU. (**C**) Survival curves of mice in the bacteremia model (*n* = 8). The model group mice received intravenous injection of *S. pneumoniae* (2 × 10^8^ CFU/mouse) with 100% mortality. The control group was injected with an equivalent volume of sterile PBS, and none of the mice died. Data are presented as mean ± SD of three independent experiments. One-way analysis of variance with Dunnett’s multiple-comparison test was used to determine significance (**A and B**). Panel C is representative of three independent experiments. Statistical significance was determined with the log-rank (Mantel-Cox) test. *P < 0.05, ***P < 0.001, ****P < 0.0001. ns, not significant. PLY, pneumolysin. *S. pneumoniae*, *Streptococcus pneumoniae.*

In the toxin hemolysis model, PLY exhibited potent hemolytic activity at 6.25 ng, equivalent to the positive control (Fig. 1A). In the *in vitro* hemolysis model of *S. pneumoniae*, the minimum complete hemolytic dose was 3 × 10^7^ CFU (Fig. 1B). In the murine bacteremia model, mice in the model group were injected intravenously with 200 µL phosphate-buffered saline (PBS) containing *S. pneumoniae* (2 × 10^8^ CFU per mouse), resulting in 100% mortality. In contrast, no mortality was observed in PBS-injected controls (Fig. 1C).

### Chimeric antibody combined with PLY protects RBCs and mice infected with *S. pneumoniae*

**Fig 2:**
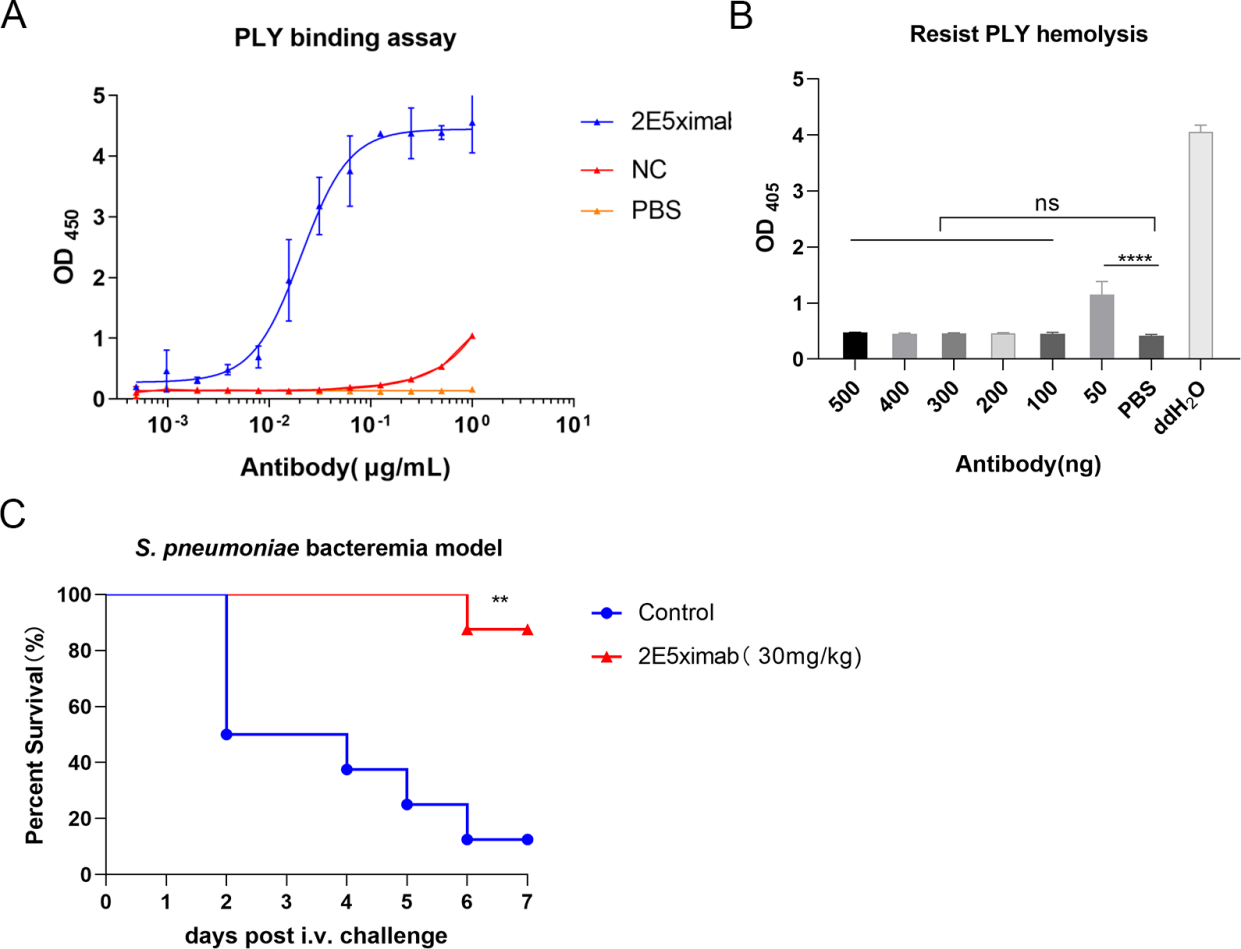
Protective effects of 2E5ximab *in vitro* and *in vivo*. (**A**) Affinity of 2E5ximab for PLY detected by ELISA. EC_50_ was 0.02063 µg/mL. NC was the negative control, and PBS was the blank control. (**B**) PLY hemolysis inhibition by 2E5ximab. PBS served as a negative control, while ddH_2_O was a positive control. (**C**) Survival curves of *S. pneumoniae*-infected mice (*n* = 8). Mice received 30 mg/kg IgG (control group) or 2E5ximab (2E5ximab group) 1 h pre-infection (2 × 10^8^ CFU/mouse). Survival was monitored for 7 days. Data shown in panels A and B are the mean ± SD of three independent experiments. One-way analysis of variance with Dunnett’s multiple-comparison test was used to determine significance (B). Panel C is representative of three independent experiments. Statistical significance was determined with the log-rank (Mantel-Cox) test. **P < 0.01, ****P < 0.0001. ns, not significant.

Building on antibody engineering principles for humanization, we designed the chimeric antibody 2E5ximab by retaining the murine variable region and replacing the constant region with human sequences (25). We assessed the *in vitro* affinity of 2E5ximab for PLY protein using enzyme-linked immunosorbent assay (ELISA) and observed a favorable affinity with an EC_50_ value of 0.02063 µg/mL (Fig. 2A). Additionally, we evaluated the anti-hemolytic activity of 2E5ximab in an *in vitro* model by co-incubating 6.25 ng of PLY protein with varying concentrations of 2E5ximab for 10 min, followed by the addition of an equal volume of 5% rabbit erythrocytes and incubation at 37°C for 1 h. The results indicated that 2E5ximab exhibited partial anti-hemolytic activity at 50 ng and complete anti-hemolytic effect at 100 ng (Fig. 2B). Furthermore, the protective efficacy of 2E5ximab against *S. pneumoniae* bacteremia was tested in murine models. Mice administered 2E5ximab (30 mg/kg) 1 h before infection exhibited a significant increase in survival rate (*P* < 0.01), indicating potent prophylactic efficacy against pneumococcal challenge (Fig. 2C).

### 2E5zumab binding with PLY exhibits anti-hemolytic activity *in vitro*

**Fig 3:**
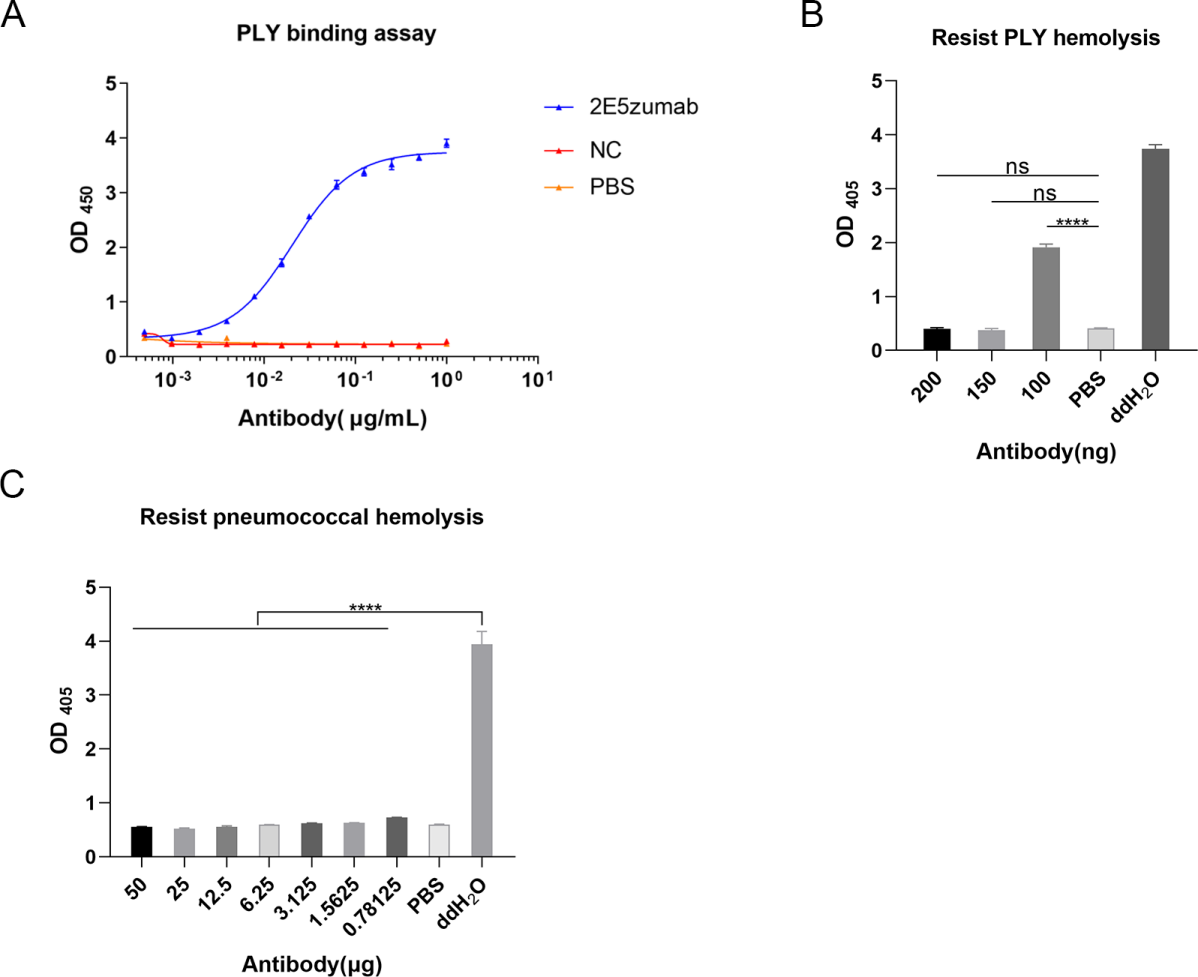
Protective effects of 2E5zumab *in vitro*. (**A**) Affinity of 2E5zumab for PLY detected by ELISA. EC_50_ was 0.02034 µg/mL. NC was a negative control, and PBS was a blank control. (**B**) Inhibition of PLY hemolysis by 2E5zumab. (**C**) Inhibition of pneumococcal hemolysis by 2E5zumab. PBS served as a negative control, while ddH_2_O was a positive control (**B and C**). Data are presented as mean ± SD of three independent experiments (**A, B and C**). One-way analysis of variance with Dunnett’s multiple-comparison test was used to determine significance (**B and C**). ****P < 0.0001. ns, not significant.

Based on the chimeric antibody, the humanized antibody 2E5zumab was engineered through complementarity-determining region (CDR) grafting and framework back mutations (25). ELISA binding assays demonstrated a high-affinity interaction between 2E5zumab and PLY with an EC_50_ value of 0.0203 µg/mL (Fig. 3A). We conducted anti-hemolysis assays and observed that 2E5zumab completely neutralized toxin activity at 150 ng (Fig. 3B). Furthermore, *in vitro* assays against *S. pneumoniae*-induced hemolysis on RBCs showed that 2E5zumab inhibited hemolysis at concentrations as low as 0.78125 µg (Fig. 3C).

### 2E5zumab provides protection in the bacteremia model

**Fig 4:**
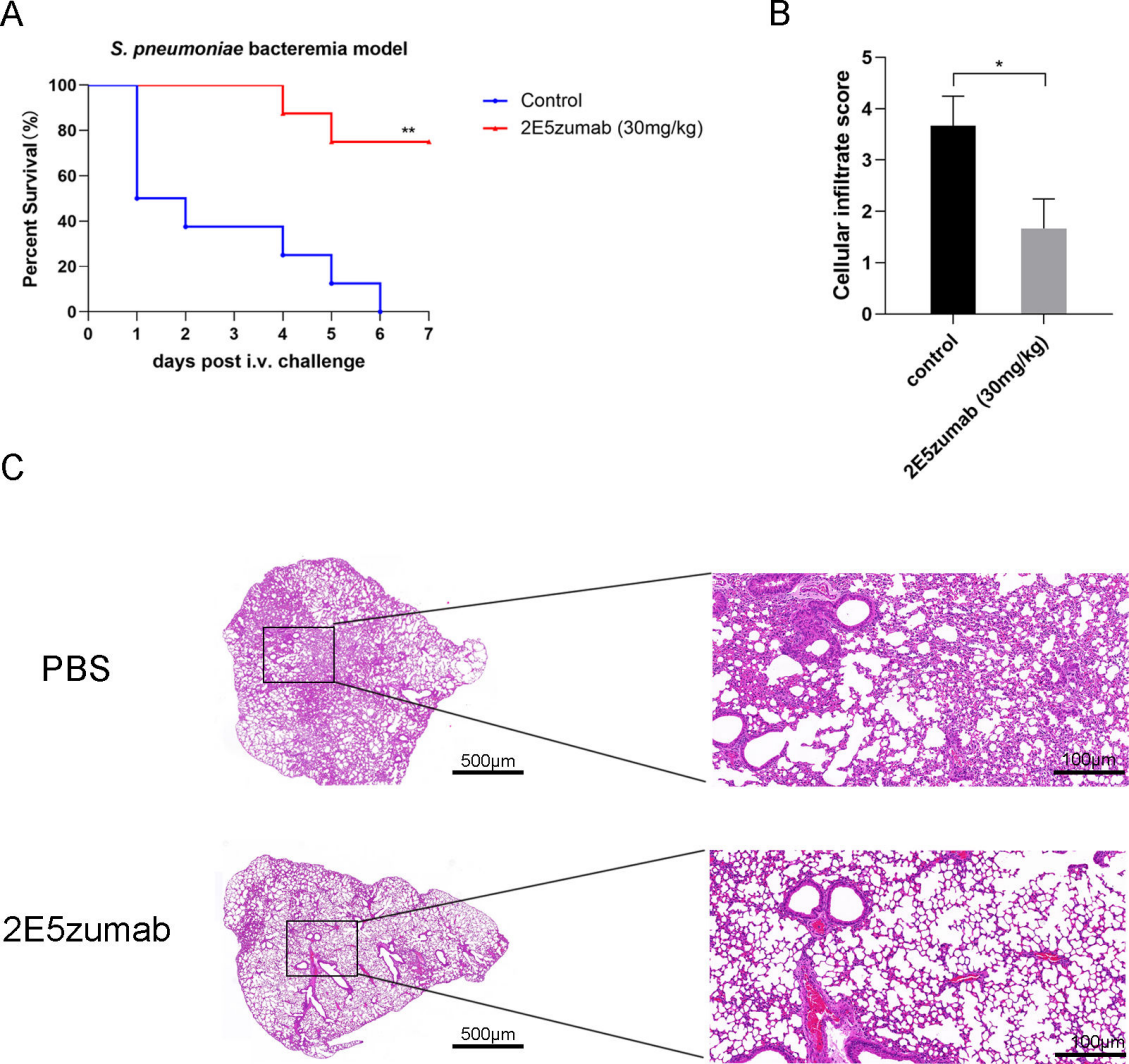
Validation of the biological activity of 2E5zumab *in vivo*. (**A**) Survival curves of *S. pneumoniae*-infected mice (*n* = 8). Mice received 30 mg/kg IgG (control group) or 2E5zumab (2E5zumab group) 1 h pre-infection (2 × 10^8^ CFU/mouse). Survival was monitored for 7 days. (**B**) Blinded H&E-stained lung sections were graded. Significantly reduced pulmonary cellular infiltration was observed in antibody-pretreated mice versus controls. Data are presented as mean ± SD of three independent experiments and analyzed by an unpaired *t*-test. (**C**) Representative H&E-stained lung photomicrographs. Scale bar, 500 or 100 µm. *P < 0.05, **P < 0.01.

The protective efficacy of 2E5zumab against *S. pneumoniae* bacteremia was evaluated in a lethal murine model. Prophylactic administration of 2E5zumab (30 mg/kg, intravenous) 1 h prior to *S. pneumoniae* challenge (2 × 10^8^ CFU/mouse) significantly improved survival rates (*P* < 0.01) (Fig. 4A). To assess lung injury, hematoxylin and eosin (H&E)-stained sections were scored for cellular infiltration 24 h post-infection. Mice pretreated with 2E5zumab exhibited markedly reduced pulmonary cellular infiltration scores compared to controls (*P* < 0.05) (Fig. 4B). Consistently, severe inflammatory cell accumulation in alveolar spaces was observed in control mice, whereas 2E5zumab pretreatment attenuated this pathology (Fig. 4C).

### Pharmacokinetic characteristics of 2E5zumab in mice

**Fig 5:**
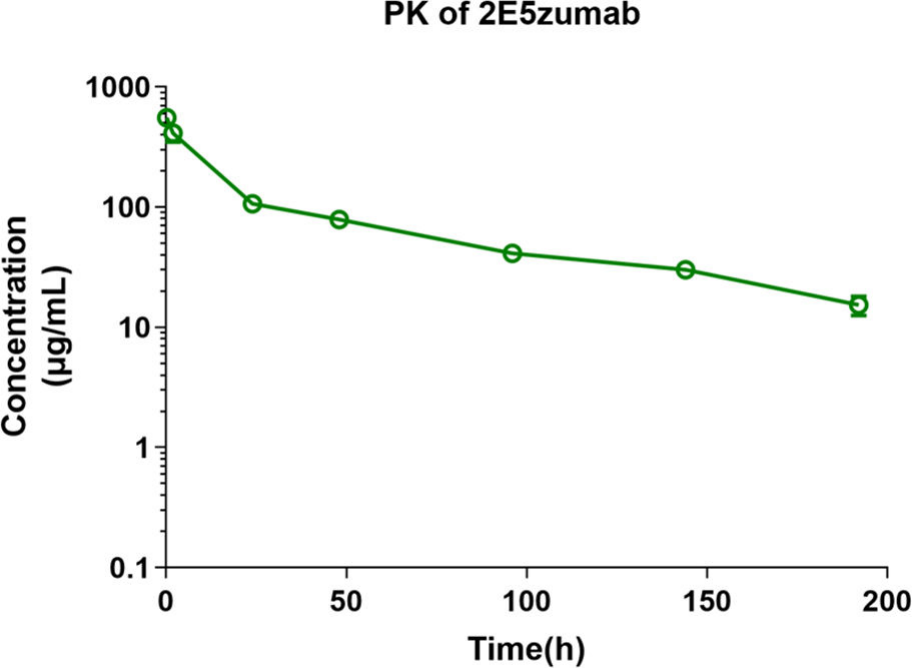
Pharmacokinetic profile of 2E5zumab in mice after single intravenous (i.v.) administration. Serum concentration-time curve of 2E5zumab following a single i.v. dose (30 mg/kg). Blood samples were collected at 0, 0.25, 2, 24, 48, 96, 144, and 192 h post-injection. Data are presented as mean ± SD of three replicates from one experiment.

The pharmacokinetic parameters of 2E5zumab were evaluated in mice following a single intravenous administration via the tail vein at a dose of 30 mg/kg. Blood samples were collected at various time points to quantify the antibody concentration in the plasma (Fig. 5). The pharmacokinetic analysis revealed that 2E5zumab was eliminated slowly from the systemic circulation of mice, exhibiting a terminal elimination half-life (*T*_1/2_) of 62.03 h. The maximum concentration (Cmax) observed was 554.86 µg/mL. The area under the concentration-time curve (AUC 0–192 h) was determined to be 14,559.38 h*μg/mL. The clearance (CL) of the drug was calculated to be 1.88 mL/h/kg, and the apparent volume of distribution (Vd) was 168.49 mL/kg (Table 1).

**TABLE 1:** Pharmacokinetic parameters of 2E5zumab (30 mg·kg^−1^)-administered tail vein in mice^*a*^

Parameters	Unit	Value
T1/2	h	62.03
Cmax	μg·mL^−1^	554.86
AUC (0–192 h）	h*μg·mL^−1^	14,559.38
AUC (0–∞）	h*μg·mL^−1^	15,934.38
Vd	mL·kg^−1^	168.49
Cl	mL·h^−1^·kg^−1^	1.88
MRT	h	46.42
Vss	mL·kg^−1^	125.59

^*a*^ T1/2: terminal elimination half-life of t1/2. Cmax: peak concentration. AUC: area under the cure. Vd: apparent volume of distribution. Cl: clearance rate. Vss: apparent volume of distribution at steady-state concentration.

## DISCUSSION

*S. pneumoniae* is an opportunistic pathogen with an alarming growing disease burden worldwide (26, 27). In 2000, about 14.5 million episodes of serious pneumococcal disease (uncertainty range 11.1–18.0 million) were estimated to occur (28). The major protein virulence factor of *S. pneumoniae*, PLY, plays a critical role in its pathogenicity (29). In this study, we developed the humanized antibody 2E5zumab, which exhibits high affinity for PLY, provides a high level of protection to the mice, and has a long *in vivo* half-life. Based on these findings, 2E5zumab holds considerable promise as an anti-pneumococcal mAb.

In recent years, the application of mAbs in respiratory diseases has made rapid progress, especially in the fields of asthma and respiratory tract infections. Tezepelumab, a human mAb that blocks thymic stromal lymphopoietin (an epithelium-derived cytokine implicated in the pathogenesis of asthma), has advanced to Phase 3 clinical trials (30). In July 2023, the first long-acting monoclonal antibody nirsevimab (Beyfortus, Sanofi, and AstraZeneca) was approved by the Food and Drug Administration (FDA) for the prevention of respiratory syncytial virus disease in infants and young children (31). Multiple mAbs have been shown to be effective for both prophylaxis and therapy for SARS-CoV-2 infection, including tixagevimab-cilgavimab (AZD7442), casirivimab and imdevimab (REGEN-COV) (32), etc. A Phase 3 trial has shown that a single dose of AZD7442 had efficacy for the prevention of coronavirus disease without evident safety concerns (33). MEDI3902 (gremubamab) is a first-in-class bivalent, bispecific human immunoglobulin G1 kappa mAb, prophylactic MEDI3902 administration protected against lethal *Pseudomonas aeruginosa* in animal models. However, a placebo-controlled Phase 2 study highlighted that MEDI3902 did not reduce *P. aeruginosa* nosocomial pneumonia incidence in *P. aeruginosa*-colonized mechanically ventilated subjects (34).

At present, mAbs against *S. pneumoniae* are still in the research stage, with no FDA-approved therapies for *S. pneumoniae.* The mAb targets of *S. pneumoniae* include the pneumococcal histidine triad protein (PhtD), pneumococcal surface protein A (PspA), capsular polysaccharides (CPSs), PLY, etc. In a mouse model of secondary pneumococcal infection, protection mediated by mAb PhtD3 and another mAb targeting a different epitope, PhtD7, was reduced. However, robust protection was restored by combining mAb PhtD3 with mAb PhtD7, indicating a synergistic effect (35). Another study showed that a panel of mouse mAb against *S. pneumoniae* serotype 3 CPS was protective both *in vivo* and *in vitro* (36). Additionally, combination therapy with antibiotics and mAbs has shown potential to enhance therapeutic outcomes. Previous studies have shown that anti-PspA antibodies enhanced the bactericidal effect of indolicidin on pneumococci (37). Adjunct treatment with pIgR and PECAM-1 antibodies to antibiotics may prevent pneumococcal meningitis development and associated brain damage (38). PLY is a major protein virulence factor of *S. pneumoniae*. The hemolytic activity of PLY, as demonstrated by our *in vitro* experiments, emphasizes its role in the pathogenesis of pneumococcal disease. Although previous studies have reported that certain mAbs targeting PLY (murine-derived antibody 6E5) demonstrated toxin-neutralizing efficacy *in vitro*, the effect *in vivo* remains to be validated (39). Since the application of mouse-derived mAbs to humans triggers an immune response to human anti-mouse antibodies (40), reducing their effects, we successfully developed a human-derived anti-*S. pneumoniae* antibody, 2E5zumab, by using the CDR grafting technology. In this study, 2E5zumab exhibited significant anti-hemolytic activity, and this anti-hemolytic activity was crucial because it directly affected host cell survival and health, mitigating one of the main mechanisms of tissue damage during infection.

In this study, we utilized a bacteremia model induced by intravenous inoculation to evaluate the systemic efficacy of antibodies. This approach ensures consistent bacterial dissemination, thereby minimizing experimental variability compared to respiratory infection models, which are inherently influenced by host-specific factors, such as mucociliary clearance and airway anatomical heterogeneity. In addition, the antibody was administered 1 h prior to *S. pneumoniae* infection to assess its prophylactic efficacy and ensure immediate immune protection. Our study demonstrated that 2E5zumab significantly improved survival rates, and notably, the histopathological analysis of lung tissues from treated mice showed a reduction in inflammatory cell infiltration, suggesting that 2E5zumab not only neutralizes the toxin but also ameliorates the inflammatory response, which is a key aspect of the pathophysiology of pneumococcal disease. While our bacteremia model provides robust insights into the systemic efficacy of antibodies, we acknowledge that respiratory infection models are better at mimicking natural disease progression. Our future work will focus on establishing respiratory infection models via intranasal or intratracheal inoculation and evaluating the synergistic effects of 2E5zumab with existing antimicrobial therapies in both bacteremia and respiratory infection models.

After confirming the pharmacodynamic effects of 2E5zumab, we conducted a preliminary pharmacokinetic study. Pharmacokinetics of 2E5zumab is characterized by a long half-life and high bioavailability. These properties support its potential clinical utility, as they enable the maintenance of sustained antibody levels in the circulation. Such sustained antibody levels are essential for providing continuous protection against toxins, thereby enhancing the effectiveness of long-term treatment strategies.

The results of this study have important implications for the development of therapeutic interventions against pneumococcal infections. The effectiveness of 2E5zumab in neutralizing PLY and providing protection against pneumococcal bacteremia suggests that antibody therapy may be an effective complement to current treatment regimens that rely primarily on antibiotics and supportive care. Although humanized 2E5zumab antibodies are promising for pneumococcal disease treatment, several limitations should be acknowledged in this study. First, 2E5zumab has high affinity for PLY protein, but the exact binding site is unknown. Second, while the bacteremia model employed in this study ensured consistent bacterial dissemination and minimized experimental variability, respiratory infection models exhibit superior capacity to mimic natural disease progression. Third, this study focuses on the preventive antibody treatment strategy rather than the post-infection treatment.

In conclusion, the humanized antibody 2E5zumab is a novel and effective approach to combat morbidity and mortality associated with pneumococcal infections. Further exploration of the synergistic effects between the antibody and existing antimicrobial therapies, combined with validation in murine respiratory infection models, could provide a comprehensive strategy for managing pneumococcal infections.

## MATERIALS AND METHODS

### Bacterial strains and cell line

*S. pneumoniae* ATCC49619 was obtained from the Laboratory of Shanghai Lung Hospital, Shanghai, China. Expi293F cells were purchased from Thermo Corporation. Moreover, 5% rabbit blood erythrocytes were purchased from Sembega Biotechnology (Nanjing, China).

### Expression and purification of PLY

The PLY protein was established by total gene synthesis by Tsingke Biotechnology (Shanghai, China), and its expression plasmid was cloned into the pET21a vectors. The resulting plasmid was transformed into *Escherichia coli* BL21(DE3) competent cells, and a monoclonal colony was selected from the transformation plate and transferred to Luria-Bertani (LB) liquid medium supplemented with 100 μg/mL of ampicillin. A 2 mL overnight culture was grown in LB plus ampicillin, then diluted 1:100 in a 100 mL culture and grown at 37°C to an OD_600_ of 0.6–0.8. Isopropyl β-D-thiogalactopyranoside (Sangon Biotech) was added to the culture to achieve a final concentration of 0.1 mM, and the culture was incubated for an additional 16 h at 16°C. The bacterial solution mixture was centrifuged at 8,000 rpm for 3 min, and the pellet was then resuspended in PBS and collected. The bacterial suspension was sonicated for three circulations of 180 s (300 W) at 0°C, and protein was collected. PLY was purified via a one-step Ni-affinity column using washing and elution buffers for the purification (41).

### Culture and preparation of bacterial fluids of *S. pneumoniae*

*S. pneumoniae* should be inoculated evenly on a Columbia blood plate and incubated at 37°C overnight. A blood agar block (2.5 × 2.5 cm) was aseptically transferred into 15 mL of sterile Todd-Hewitt broth/yeast extract medium, then incubated for 3.5–4 h at 37°C until the OD_600_ reaches 0.6–0.8. The bacterial solution mixture was centrifuged at 13,000 rpm for 5 min, and the supernatant should be discarded. It should be washed twice with PBS and resuspended with PBS.

### Expression and purification of antibodies

The nucleotide sequences of heavy and light chains of antibodies after codon optimization were synthesized and cloned into the pcDNA3.1(+) vector by Tsingke Biotechnology (Shanghai, China). Antibodies were expressed by transfection of Expi293F cells. The cell density was adjusted to 3 × 10^6^/mL and cultured in a shaking bed at 37°C. After 7 days, the cell supernatant was collected, and the antibody was purified with a protein A it.

### ELISA for affinity detection

The PLY protein was coated in the bottom of 96-well ELISA plates at a concentration of 0.1 μg/well overnight at 4°C .The following day, after blocking with 5% bovine serum albumin at 37°C for 1 h, serial twofold dilution antibodies were prepared in PBS, added into the antigen-coated plates, and incubated overnight at 4°C. On the third day, the second antibody with HRP was used, and plates were incubated for 1 h at 37°C. The 3,3′,5,5′-tetramethylbenzidine substrate was used as the substrate for color development, and plates were incubated for 4 min. The 2 M HCl was then added to stop the reaction. Finally, the reaction mixture absorbance was measured at 450 nm using a multi-mode microplate reader.

### Hemolysis model

After 5% rabbit blood was washed three times with PBS buffer, red blood cells (RBCs) were added to a 96-well plate at a volume of 100 μL/well. Different concentrations of toxins were added, and the samples were incubated at 37°C for 1 h using PBS as a negative control and ddH_2_O as a positive control. After 1 h, the samples were centrifuged at 3,000 rpm and 4°C for 1 min. The supernatant was collected, and its OD_405_ (absorbance value at 405 nm) value measured. A higher OD_405_ value indicates more severe damage to RBCs, corresponding to a toxin with a stronger hemolytic activity. To evaluate the effectiveness of antibodies that block the toxin’s hemolytic effect, the toxin and the antibody should be preincubated at 37°C for 10 min before induction of RBC injury. To assess the effectiveness of antibodies that block the hemolytic effects of *S. pneumoniae*, *S. pneumoniae* and the antibodies should be preincubated for 10 min at 37°C before inducing erythrocyte damage (42).

### Animal model

To study pneumococcal infection *in vivo*, female C57BL/6 mice aged 6–8 weeks were injected intravenously with 200 μL of PBS containing live *S. pneumoniae* (2 × 10^8^ CFU per mouse), with eight mice per group. To investigate the protective effect of antibodies in the murine bacteremia model, the antibodies were injected into the tail vein 1 h before bacterial injection at a dose of 30 mg/kg, and control mice received an equivalent dose of IgG intravenously 1 h prior to infection. Each experiment group included eight mice. Mice were provided with sufficient food and water, and survival rates were monitored over a period of 7 days. In the pathologic examination experiment, after 24 h of infection, mice were euthanized, and their lungs were harvested, fixed in 4% neutral buffered formalin, and stained for H&E. Each experiment group included three mice. Blinded lung sections were graded for cellular infiltration as follows: Grade 0 (none)—no immune cells found in the lungs; Grade 1 (mild)—focal aggregates of immune cells usually around bronchioles and blood vessels; Grade 2 (moderate)—dense cuffs of lymphocytes surrounding blood vessels and airways; Grade 3 (marked)—moderate infiltrate, extending into the surrounding tissue, mostly macrophages in alveoli and variable neutrophils and lymphocytes; and Grade 4 (severe)—marked infiltrate with extensive areas of the lung tissue affected (43).

### Pharmacokinetics

To investigate the pharmacokinetic characteristics of the antibody *in vivo*, three female C57BL/6 mice aged 6–8 weeks were administered the humanized antibody 2E5zumab (30 mg/kg) in the tail vein, and the blood samples were collected from the orbital region of the eyes of mice anesthetized with isoflurane inhalation at 0 and 15 min and 2, 24, 48, 96, 144, and 192 h, respectively. The blood samples were allowed to stand at room temperature for 30 min and centrifuged at 4°C and 9,000 rpm for 15 min, and the supernatant of the blood samples was sent to the company for the detection of blood concentration and pharmacokinetic parameters.

### Statistical analysis

Data were representative of three independent experiments, unless otherwise indicated. Software GraphPad Prism 8.0 was used to evaluate data, and data are shown as mean ± SD. In hemolysis and anti-hemolysis tests, one-way analysis of variance with Dunnett’s multiple-comparison test was used to determine significance. The cellular infiltrate score in histopathological analysis was compared using a two-tailed Student’s *t*-test. A log-rank test was used to evaluate the survival curve equality. A *P* value of < 0.05 was considered statistically significant.
